# Selective Sorption of Gold and Iron Ions in the PMAA-P4VP Intergel System

**DOI:** 10.3390/molecules31101583

**Published:** 2026-05-09

**Authors:** Talkybek Jumadilov, Gulnur Dyussembayeva, Zhazira Mukatayeva, Juozas Gražulevicius, Khuangul Khimersen

**Affiliations:** 1 Laboratory of Synthesis and Physicochemistry of Polymers, A.B. Bekturov Institute of Chemical Sciences JSC, 106 Sh. Valikhanov Street, Almaty 050010, Kazakhstan; jumadilov_kz@mail.ru (T.J.); huana88@mail.ru (K.K.); 2 Institute of Natural Sciences and Geography, Abai Kazakh National Pedagogical University, 13 Dostyk Ave., Almaty 050010, Kazakhstan; jazira-1974@mail.ru; 3 Department of Polymer Chemistry and Technology, Kaunas University of Technology, 73 K. Donelaičio Street, 44249 Kaunas, Lithuania; juozas.grazulevicius@ktu.lt

**Keywords:** intergel system, ion exchangers, hydrogels, PMAA, P4VP, remote interactions, sorption, gold ions, iron ions, mutual activation

## Abstract

This study presents a method for the efficient separation of gold and iron ions using cross-linked polyelectrolyte interpolymer systems. The research aims to investigate the sorption behavior resulting from the remote interaction of intergel systems based on polymethacrylic acid (gPMAA) and poly-4-vinylpyridine (gP4VP) hydrogels. According to the atomic emission spectroscopy results, the individual gPMAA (6:0) hydrogel exhibits negligible activity toward gold ions, with a sorption degree of only 5.97%, while maintaining a high sorption efficiency for iron ions at approximately 90.27%. The interpolymer interaction effect is most pronounced in the equimolar (3:3) intergel system, where the sorption efficiency for gold ions significantly increases, reaching a peak of 63.97% after 24 h of contact. In contrast, the sorption efficiency for iron ions remains consistently high across all molar ratios, fluctuating between 88.27% and 91.33%. The polymer chain binding degree (θ) further confirms these trends, reaching maximum values of 1.09% for gold and 7.18% for iron ions. These findings demonstrate that the gPMAA-gP4VP system possesses a high selective affinity for iron over gold under the studied conditions, providing a viable approach for the selective separation and recovery of metals from multicomponent industrial solutions.

## 1. Introduction

Currently, the global reserves of gold-bearing ores are declining annually. Due to a growing demand for gold, the gold mining companies are forced to process low-grade ores, using various new technologies. According to the sources, the studied mining companies in Kazakhstan possess significant gold reserves, sufficient for a stable operation for over 50 years at the current production rates [1]. Gold, along with silver, platinum, and other elements (ruthenium, rhodium, palladium, osmium, and iridium), is one of the noble metals. It is designated by the symbol “Au”, which comes from the Latin word “aurum” [2]. Gold mining is a complex and expensive process that requires the use of modern methods and technologies [3]. Extracting gold from ore is a critical task in the precious metals production, and modern methods and technologies are used to optimize and improve this process. One of the most widely used methods is heap leaching, which extracts gold from ore through chemical reactions. The use of modern gold mining technologies has a negative impact on the environment. Therefore, the development and implementation of environmentally friendly gold mining methods is a significant challenge for the raw materials industry.

Heap leaching is a widely adopted method for extracting precious metals by treating ore heaps with specialized chemical solutions. Currently, it is one of the most common techniques for processing gold-bearing low-grade ores, allowing for the expansion of the raw material base and improving the economic performance of mining operations [4]. Today, heap leaching is utilized by approximately 60% of the world’s leading gold-producing countries due to its efficiency and cost-effectiveness. However, a significant drawback of this method is its environmental impact, as potassium or sodium cyanide is typically used as the primary leaching agent [5]. The use of these cyanide solutions leads to the formation of highly stable dicyanoaurate complexes, [Au(CN)_2_]^−^, enabling efficient gold dissolution. Despite its efficacy, the process is often complicated by the co-extraction of iron, which forms various cyanide complexes that compete for active sites during recovery. Alkaline cyanide leaching systems typically provide around 73% gold dissolution compared to other solvents [6]. Consequently, the development of selective ion-exchange materials for the effective separation of gold from multicomponent cyanide leachates remains a critical challenge for the industry.

Cross-linked polymers, or hydrogels, are chemically interconnected networks that possess unique physicochemical properties. These materials are characterized by a high density of ionizing groups along their macrochains, which imparts specific electrochemical, hydrodynamic, and conformational behaviors to the system [7,8]. Ion-exchange resins are artificially created high-molecular-weight solids with ionogenic groups, capable of exchanging soluble electrolyte ions. Ion-exchange resins are used for the sorption, using durable resins with functional groups that reliably bind metal ions. In modern society, the potential for synthesizing new polymeric materials, such as polymer complexes of hydrogels and ion exchangers with a high conductivity, has attracted the attention of researchers [9,10]. The physicochemical properties of hydrogels are determined by the specific nature of the functional groups they contain. For several years, particular attention has been paid to hydrogels capable of forming the inter- and interpolymer complexes in the presence of cross-linked linear macromolecules [11,12]. Recent advancements in 2025 have further pushed the boundaries of these systems; for instance, ionic phosphine-functionalized porous organic polymers have demonstrated rapid and highly selective Au(III) recovery [13], while solution-processed porous polymers have achieved unprecedented adsorption capacities [14]. Moreover, the integration of ion-imprinted polymer coatings on magnetic supports has proven effective for atomic-level selectivity in multi-metal recovery environments [15].

In the course of the systematic studies of the interaction of hydrogels with various compounds, changes in their properties have been observed during the long-range interaction of two hydrogels of different natures. For some salts, the order of properties of the intergel systems at certain molar ratios has been considered [16,17]. The results of the study have shown that hydrogel interactions occur through long-range interactions, resulting in the hydrogels becoming highly ionized, and the electrochemical properties of the solutions, such as conductivity and pH, undergoing significant changes. Recent studies have shown that the interpolymer systems are valuable candidates for the sorption technology [18,19]. The sorption capacity of the interpolymer systems is higher than that of individual polymers [20]. Therefore, in our study, hydrogels of polymethacrylic acid (PMAA) and poly-4-vinylpyridine (P4VP) have been selected to form an intergel pair as a result of the long-range interaction in the PMAA-P4VP intergel system [21].

Polymethacrylic acid (PMAA) is a synthetic, water-soluble polymer obtained by the polymerization of methacrylic acid. It is a colorless, brittle, and highly hygroscopic material that does not undergo a highly elastic state or melt upon heating. Due to its unique physicochemical properties, PMAA is widely utilized in the production of copolymers, household chemicals, and various applications within the food industry. It is used in combination with acrylic esters, styrene, butadiene, and other monomers to create various materials [22,23].

Poly-4-vinylpyridine (P4VP) is a polymer formed by the polymerization of the monomer 4-vinylpyridine, which contains a vinyl group (C=C) and a pyridine ring in its structure. It has a wide range of applications and often acts as a polyelectrolyte. Its chemical properties can be modified by introducing additional chemical groups into its structure [24].

The main objective of this study is to investigate and prove the sorption capacity of the intergel systems based on polymethacrylic acid (gPMAA) and poly-4-vinylpyridine (gP4VP) in relation to gold ions.

## 2. Results

### 2.1. Sorption Efficiency (η) of Gold and Iron Ions by the Interpolymer System

**Figure 1:**
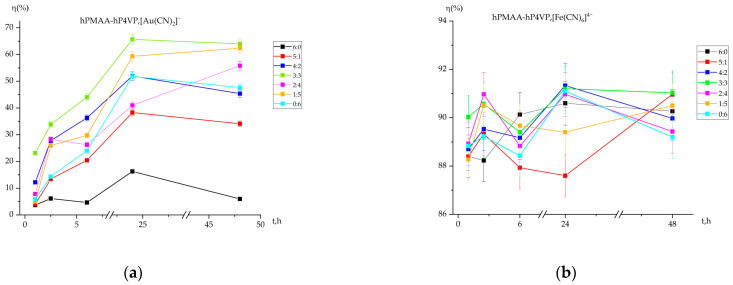
Dependence of the sorption efficiency (η) of Au(I) (**a**) and Fe(II) (**b**) ions on the contact time and molar ratio of PMAA-P4VP intergel systems. Experimental conditions: C(Au(I)) = C(Fe(II)) = 30 mg/L; solution volume = 200 mL; sorbent dose = 0.12 g; temperature = 25 ± 1 °C; pH maintained at 6.97.

Previous studies have established that interpolymer systems based on polymethacrylic acid (PMAA) and poly-4-vinylpyridine (P4VP) exhibit high potential for metal extraction from model solutions. These findings demonstrated that sorption efficiency depends significantly on polymer composition and experimental conditions, with the system maintaining structural integrity for repeated use [25,26]. Building on these premises, the sorption efficiency in the current study was evaluated to identify the optimal parameters for metal recovery. The results confirm that the degree of sorption serves as a critical kinetic parameter, highly sensitive to the molar ratio of the interacting hydrogels. The extraction of gold ions (Figure 1a) exhibits a pronounced induction period at the initial stages, followed by a rapid increase in efficiency after 6 and 24 h of contact. Individual PMAA (6:0) shows the lowest activity, whereas the introduction of P4VP significantly enhances the process. The highest sorption degree was recorded for the equimolar system (3:3) and the polybase-rich system (1:5), reaching 65.67% and 62.37%, respectively. This trend confirms that the presence of P4VP units provides essential active sites for [Au(CN)_2_]^−^ complexation. The stabilization of the sorption degree after 24 h suggests that the system achieves a state of maximum saturation, where the “long-distance interaction” between the networks is fully realized.

In contrast to gold, the sorption efficiency for iron ions remains consistently high across all ratios, fluctuating within a narrow range of 88.27% to 91.33%. This indicates a high affinity of the PMAA-P4VP intergel system toward [Fe(CN)_6_]^4−^ complexes. The maximum efficiency was observed at the 4:2 and 3:3 ratios after 24 h of interaction, reaching 91.30% and 91.20%, respectively. The kinetic curves for iron ions (Figure 1b) show a characteristic “wave-like” behavior during the first 6 h, which is likely associated with the sequential protonation/deprotonation of the polymer chains and subsequent conformational adjustments of the intergel network. After 24 h, the system reaches a stable plateau, proving the high efficiency and reliability of these ratios for iron ion recovery.

A comparative analysis shows that the PMAA-P4VP system is more effective for iron ion sorption than for gold, although both metals show peak recovery at the 3:3 equimolar ratio. This confirms that the mutual activation effect arising from the interaction of two chemically different hydrogels—a polyacid and a polybase—creates a more effective sorption environment than individual polymer components. These findings substantiate the potential of intergel systems for the selective treatment of multicomponent industrial solutions. Analysis of the kinetic data shows that the sorption process reaches its active peak within 24 h of interaction. The slight decrease in sorption efficiency observed at 48 h indicates a transition toward a long-term stable equilibrium. This phenomenon can be explained by the structural reorganization of the PMAA-P4VP intergel system; as the polymer chains achieve a more compact conformational state, a minor redistribution of ions may occur, leading to small fluctuations in the residual concentration. Thus, while 24 h is sufficient for the primary recovery, the 48 h mark represents the final stabilization of the dynamic equilibrium within the interpolymer matrix.

### 2.2. Polymer Chain Binding Degree (θ) of Gold Ions by the Interpolymer System

**Figure 2:**
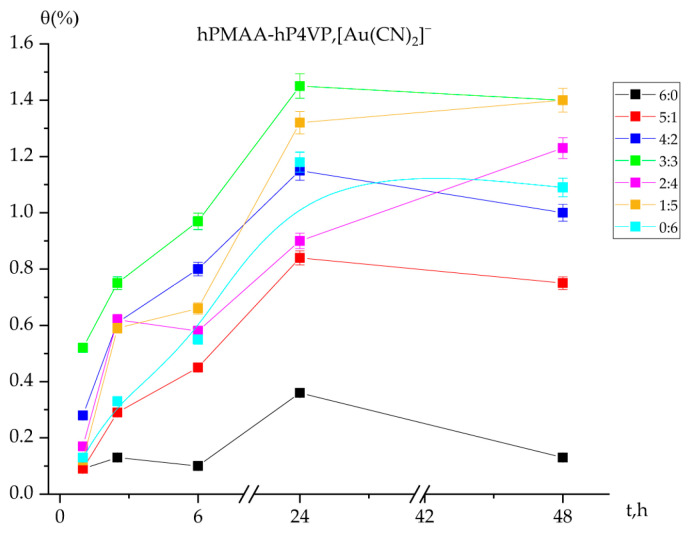
Degree of polymer chain binding of Au(I) ions by the intergel system PMAA-P4VP as a function of time. Experimental conditions: C(Au(I)) = C(Fe(II)) = 30 mg/L; solution volume = 200 mL; sorbent dose = 0.12 g; temperature = 25 ± 1 °C; and pH maintained at 6.97.

Figure 2 illustrates the degree of polymer chain binding of Au(I) ions by the intergel system PMAA-P4VP as a function of time.

The individual PMAA hydrogel (6:0) exhibited the lowest and most stable binding degree (peak θ ≈ 0.38%), which can be attributed to the lack of specific affinity between the polyacid carboxylic groups and the gold cyanide anions. In contrast, the individual P4VP hydrogel (0:6) showed a systematic increase in θ, reaching equilibrium after 24 h. This confirms that the pyridine rings of P4VP are the primary binding sites for [Au(CN)_2_]^−^ ions. The intergel systems demonstrated a markedly higher binding efficiency compared to the individual polymers. The maximum values were observed at the 3:3 and 4:2 molar ratios after 24 h of interaction. At the equimolar ratio (3:3), the system achieves an optimal conformational state that maximizes the availability of active binding sites, resulting in the highest θ values recorded. In all studied systems, the binding process begins slowly within the first 0.5–1 h (initial values around 0.09–0.25%), representing the diffusion-limited stage of the interaction. A significant growth phase is observed between 6 and 24 h, followed by stabilization at the 48 h mark. This stabilization suggests that the polymer chains have reached a saturation threshold relative to the concentration of gold ions in the solution.

### 2.3. Polymer Chain Binding Degree (θ) of Iron Ions by the Interpolymer System

**Figure 3:**
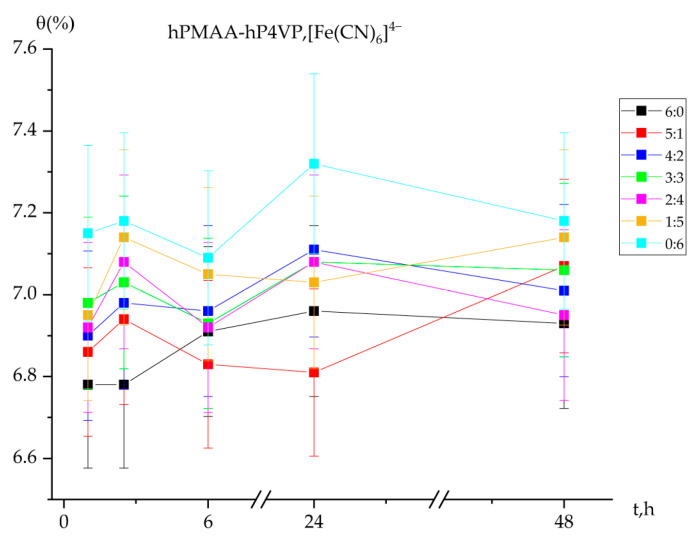
Degree of polymer chain binding of Fe (II) ions by the intergel system PMAA-P4VP as a function of time. Experimental conditions: C(Au(I)) = C(Fe(II)) = 30 mg/L; solution volume = 200 mL; sorbent dose = 0.12 g; temperature = 25 ± 1 °C; and pH maintained at 6.97.

The degree of polymer chain binding for iron ions [Fe(CN)_6_]^4−^ demonstrates a higher numerical range (approx. 6.78–7.32%) compared to gold ions, suggesting a stronger interaction between the ferrocyanide complexes and the intergel matrix. The binding capacity is notably higher in systems where poly-4-vinylpyridine (P4VP) is the dominant component. As shown in Figure 3, the individual P4VP hydrogel (0:6) achieved a maximum binding degree of 7.32% after 24 h. In contrast, the individual PMAA hydrogel (6:0) remained relatively stable with minimal fluctuations, confirming that the carboxylic groups of the polyacid possess a lower affinity for these complex anions compared to the pyridine units of the polybase.

The 4:2 and 3:3 molar ratios reached their peak binding values (up to 7.11%) after 24 h. At the 2:4 ratio, the binding degree remained constant initially (7.08%) but showed a slight decrease after 48 h to 6.95%, which may indicate the establishment of a desorption equilibrium or localized structural compaction of the gels. During the first hour of interaction, the binding degree across all systems remained relatively low (6.78–7.15%), as the sorption process had not yet reached its full activation phase. However, as the “remote effect” between the polyacid and polybase intensified, the binding degree stabilized between 6.93% and 7.18% by the 48 h mark.

### 2.4. Comparative Analysis of Sorption Efficiency and Polymer Chain Binding

**Figure 4:**
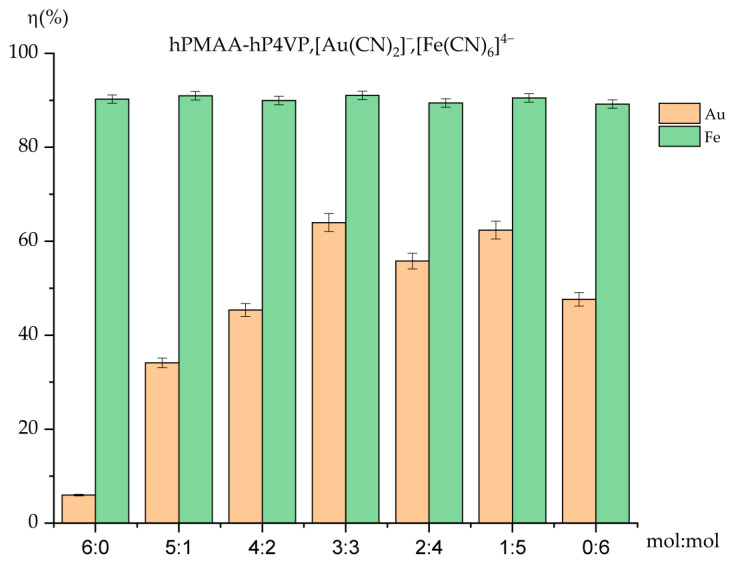
Comparative chart of competitive sorption of Au(I) and Fe(II) ions by the intergel system PMAA-P4VP. Experimental conditions: C(Au(I)) = C(Fe(II)) = 30 mg/L; solution volume = 200 mL; sorbent dose = 0.12 g; temperature = 25 ± 1 °C; and pH maintained at 6.97.

The evaluation of the sorption efficiency (Figure 4) reveals a significant disparity between the uptake of iron and gold ions by the PMAA-P4VP intergel system. Across all molar ratios, the sorption of iron ions [Fe(CN)_6_]^4−^ remains consistently high, ranging from 90.27% to 91.03%. Even in the individual PMAA (6:0) and P4VP (0:6) hydrogels, the recovery rates stay near 90%, suggesting a strong thermodynamic affinity of the polymer matrix toward these complex anions regardless of the specific gel composition.

In contrast, the sorption of gold ions [Au(CN)_2_]^−^ is more sensitive to the intergel ratio. While individual PMAA (6:0) shows negligible activity (5.97%), the equimolar 3:3 ratio demonstrates a pronounced sorption peak at 63.97%, resulting from the mutual activation of the polymer networks. This highlights that the long-range interaction between the polyacid and polybase is critical for creating an environment suitable for gold recovery. The observed peak at the 3:3 molar ratio can be explained by the electrochemical equilibrium established between the polyacid (gPMAA) and polybase (gP4VP) components. According to the theory of remote interaction in intergel systems, the equimolar ratio facilitates the optimal transfer of low-molecular-weight ions (H^+^ and OH^−^), which maximizes the effective charge density on the polymer chains. This mutual activation leads to a higher concentration of dissociated functional groups compared to individual gels or non-equimolar ratios, thus enhancing the complexation capacity of the system toward precious metal anions.

**Figure 5:**
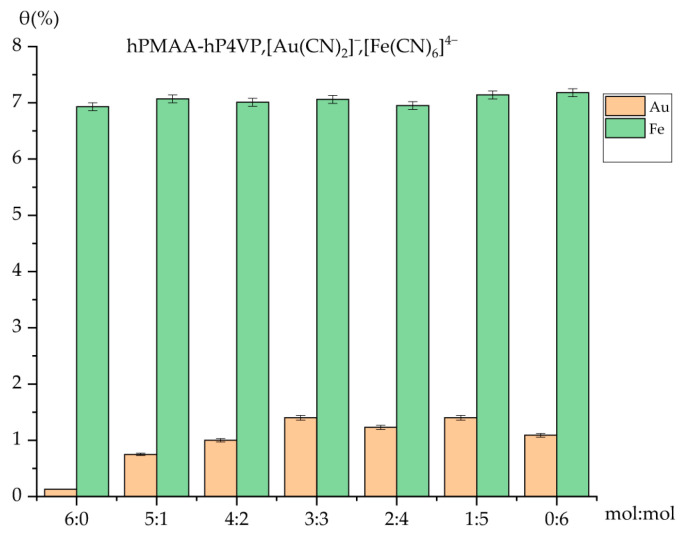
Comparison of binding degrees by the intergel system PMAA-P4VP. Experimental conditions: C(Au(I)) = C(Fe(II)) = 30 mg/L; solution volume = 200 mL; sorbent dose = 0.12 g; temperature = 25 ± 1 °C; and pH maintained at 6.97.

The binding degree further illustrates the interaction intensity (Figure 5). The results clearly show that the polymer chains are much more active in binding iron ions compared to gold. The degree of binding for iron ions is remarkably stable and high, fluctuating between 6.93% and 7.18%. This high occupancy of the polymer’s functional sites by iron ions indicates a robust complexation process that reaches near-maximum capacity in almost all studied ratios.

For gold ions, the binding degree is significantly lower, ranging from a mere 0.13% to 1.09%. The peak value (1.09%) observed in the P4VP-rich systems confirms that the pyridine nitrogen atoms are the primary, albeit limited, binding sites for the [Au(CN)_2_]^−^ complexes.

The comparative study proves that the PMAA-P4VP intergel system possesses a higher selective affinity for iron ions over gold ions under the experimental conditions. The high and stable binding parameters for iron ions (7.07–7.18%) versus the relatively weak and ratio-dependent binding of gold ions suggest that the ferrocyanide anions interact more effectively with the protonated sites of the intergel network. These findings are crucial for understanding the behavior of the system in multicomponent industrial solutions where selective metal extraction is required.

The selectivity of the gPMAA-gP4VP intergel system can be further contextualized by comparing it with recent advancements in biopolymer-based sorbents. For instance, James et al. [27] developed a chitosan-thioglycolic acid composite that exhibits high selectivity for Au(III) recovery from electronic waste via adsorption-coupled reduction. While such functionalized biopolymers show excellent performance in acidic leachates, our intergel system provides a specialized solution for alkaline cyanide media. The ‘mutual activation’ effect between gPMAA and gP4VP networks ensures that the system remains effective in the presence of competing iron cyanide complexes, offering a robust alternative for industrial gold processing.

To quantitatively evaluate the sorption capacity of the intergel system, the distribution coefficients (Kd) and separation coefficients (β) for Au(I) and Fe(II) ions were calculated and are presented in Table 1. The obtained data reveals that the PMAA-P4VP system exhibits a significantly higher affinity for iron ions [Fe(CN)_6_]^4−^ across all molar ratios, as evidenced by Kd_Fe_ values that are several times higher than the corresponding Kd_Au_ values. Specifically, at the 3:3 equimolar ratio, the distribution coefficient for iron reached its peak at 16,920.7 mL/g, while the value for gold ions remained considerably lower at 2958.7 mL/g. The separation coefficients (β = D_Au_/D_Fe_) reported in Table 1 range from 0.0068 to 0.1749, further confirming the system’s high selectivity for iron over gold ions.

### 2.5. Fourier Transform Infrared (FTIR) Spectroscopy Studies

**Figure 6:**
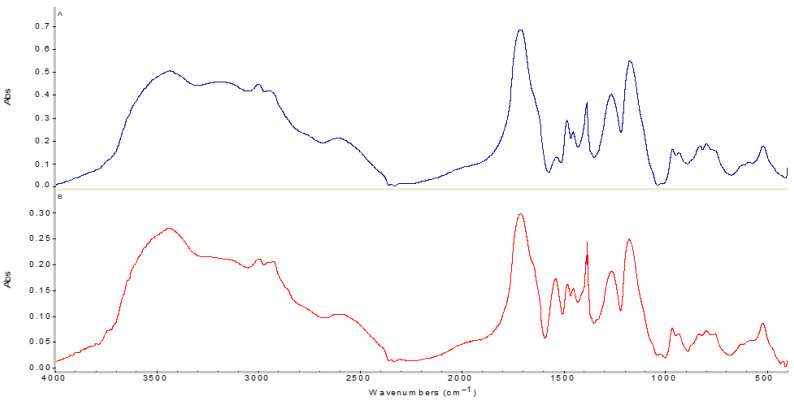
FTIR spectra of (**A**) the initial PMAA and (**B**) the PMAA component within the PMAA–P4VP intergel system (3:3 ratio) after the sorption of gold and iron ions.

To elucidate the mechanism of ion–polymer interaction, FTIR spectroscopy was employed. The analysis focused on the initial polymers and the PMAA–P4VP intergel system (3:3 ratio) after the uptake of gold and iron ions. Absorption bands in the high-energy region (3439.9 cm^−1^ and 3196.4 cm^−1^ confirm the presence of hydroxyl (-OH) groups and hydrogen-bonded water molecules, as shown in Figure 6. In the intergel system after sorption, the peaks at 2997.8 cm^−1^ and 2929.5 cm^−1^, corresponding to C–H stretching vibrations of methyl and methylene groups, remain prominent [28,29]. Strong signals at 1712.0 cm^−1^ and 1712.4 cm^−1^ are characteristic of C=O stretching in carboxylic groups. The shift and intensity change in the absorption bands at 1384.9 cm^−1^, 1481.7 cm^−1^, and 1540.6 cm^−1^ indicate the asymmetric vibrations of carboxylate groups (COO-). This confirms the active participation of PMAA carboxylic units in the interaction with metal ions. New or shifted bands at 1263.4 cm^−1^ and 1177.7 cm^−1^ suggest coordination affecting the C–O and C–N bonds [30], pointing to the involvement of P4VP pyridine nitrogen atoms in the formation of stable complexes with gold and iron. The appearance of absorption bands in the lower frequency region (421.6 cm^−1^) is indicative of structural vibrations associated with the presence of heavy metal atoms within the polymer network.

**Figure 7:**
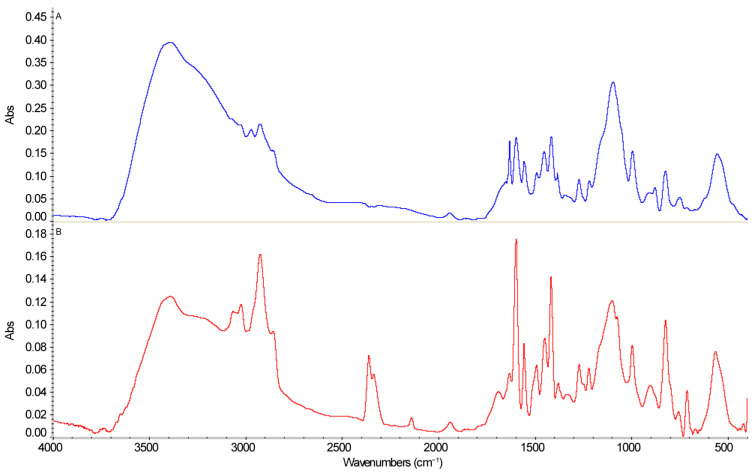
FTIR spectra of (**A**) the initial P4VP and (**B**) the P4VP component within the PMAA–P4VP intergel system (3:3 ratio) after the sorption of gold and iron ions.

In Figure 7, the FTIR spectra of the P4VP component within the PMAA–P4VP intergel system are compared before and after sorption to identify structural changes induced by gold and iron ions. The absorption bands at 3392.6 cm^−1^ and 2500 cm^−1^ are attributed to the stretching vibrations of hydroxyl groups (υOH), while the peaks at 2972.3 cm^−1^ and 2926.6 cm^−1^ correspond to the CH_2_ and CH_3_ stretching vibrations [28,29,30]. A significant change after sorption (spectrum b) is the appearance of a new distinct band at 2140.4 cm^−1^. This peak is highly characteristic of the stretching vibrations of coordinated nitrile groups (–C≡N) from the [Au(CN)_2_]^−^ or [Fe(CN)_6_]^4−^ complexes, confirming the successful formation of metal–ligand coordination bonds within the polymer matrix. Shifts observed at 1220.5 cm^−1^, 1074.1 cm^−1^, and 563.5 cm^−1^ indicate a redistribution of electron density and structural reorganization involving C–N and C–O bonds, as well as interactions with heavy metal atoms.

The FTIR spectroscopic results demonstrate that the interaction with gold and iron ions leads to stable chemical changes in the anion exchanger’s structure. The emergence of the nitrile band at 2140.4 cm^−1^ provides direct evidence of the effective coordination of metal complexes, which fundamentally determines the high efficiency and selectivity of the PMAA–P4VP intergel system.

### 2.6. Proposed Sorption Mechanism

To summarize the findings from the kinetic studies and FTIR analysis, a sorption mechanism is proposed. The interaction between the PMAA-P4VP intergel system and the metal cyanide complexes occurs through several pathways.

In an aqueous environment, the pyridine nitrogen atoms of the P4VP component undergo protonation, creating positively charged centers. These centers interact with the anionic metal complexes through electrostatic forces, effectively acting as an ion-exchange process:

R–C_5_H_4_NH^+^ + [Au(CN)_2_]^−^ ↔ R–C_5_H_4_NH^+^ · [Au(CN)_2_]^−^

4(R–C_5_H_4_NH^+^) + [Fe(CN)_6_]^4−^ ↔ (R–C_5_H_4_NH^+^)_4_ · [Fe(CN)_6_]^4−^

Furthermore, the FTIR results, specifically the emergence of the band at 2140.4 cm^−1^, confirm that the sorption is not purely electrostatic. It also involves the formation of coordination bonds between the lone pair of the nitrogen atoms in the P4VP units and the metal center, or through bridge bonding with the cyanide ligands. The PMAA component, through its carboxylic groups, contributes to the overall stability of the intergel network via hydrogen bonding, which maintains the optimal conformation for ion accessibility.

### 2.7. Thermogravimetric and Differential Scanning Calorimetric Analyses of the Intergel System PMAA-P4VP (3:3) and Its Complexes

**Figure 8:**
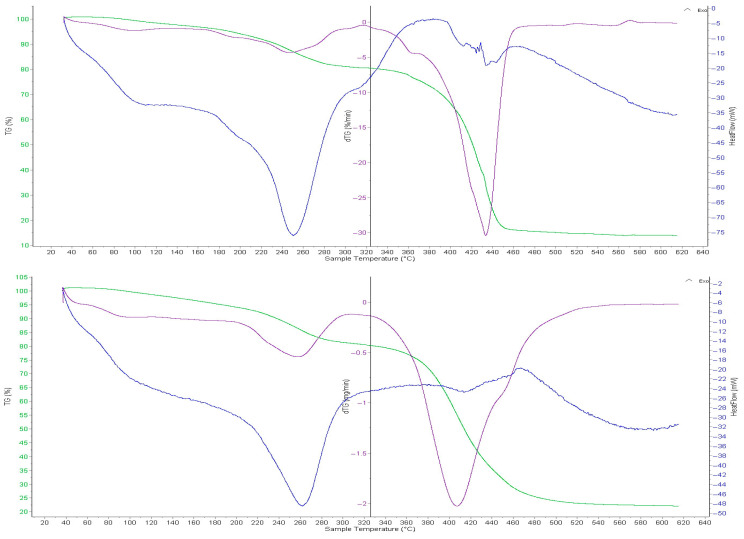
TGA and DTG curves of the initial PMAA (curve 1) and the PMAA component within the PMAA–P4VP intergel system (3:3 ratio) after the sorption of gold and iron ions (curve 2). The green line represents the TG curve, the purple line represents the DTG curve, and the blue line represents the DSC (Heat flow) curve.

In Figure 8, the thermal stability of the gPMAA sorbent samples before and after the sorption of gold and iron ions has been studied using the thermogravimetric (TG), differential thermogravimetric (DTG), and differential thermal analysis (DTA). The thermal analysis of the initial gPMAA sample (curve 1) has shown that the sample loses weight in several stages as the temperature increases. The TGA curve shows a slight decrease in weight in the temperature range from 20 to 120 °C, which is explained by the release of the adsorbed moisture and light volatile substances from the sample. In the range from 120 to 250 °C the decomposition of the weakly bound groups in the structure begins, and the mass gradually decreases by approximately 2–4%. The main decomposition of the polymer structure occurs at a temperature of 250–350 °C. A sharp decrease in mass is observed. The maximum rate of mass loss on the DTG curve is observed in the temperature ranges of 250–300 °C and 400–450 °C, where deep thermal decomposition of the polymer (visible as peaks) occurs, leading to the formation of gaseous products. The DTA confirms the thermal effects occurring in these temperature ranges, initially showing the moisture release and then degradation of the polymer structure. Regarding the thermal decomposition of gP4VP after the sorption (curve 2), a slight shift in the mass loss stages and an increase in the residual mass are observed in the thermal curves of the sorbent.

This is explained by the presence of the sorbed substances in the sorbent structure and the relative stabilization of its structure. As a result, the thermal stability of the sorbed sample is slightly increased compared to the original sorbent. The main thermal decomposition of the gPMAA sorbent occurs in the temperature range of 250–450 °C, and the sorption process to a certain extent influences the thermal stability of the material.

**Figure 9:**
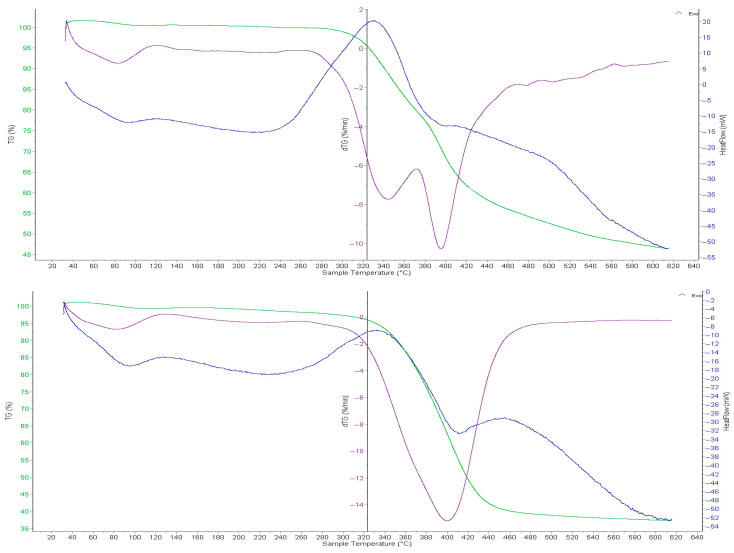
TGA and DTG curves of the initial P4VP (curve 1) and the P4VP component within the PMAA–P4VP intergel system (3:3 ratio) after the sorption of gold and iron ions (curve 2). The green line represents the TG curve, the purple line represents the DTG curve, and the blue line represents the DSC (Heat flow) curve.

Figure 9 shows the thermal degradation curves of the original P4VP (with gold and iron ions). As can be seen from the Figure 9, the curves show the change in mass upon heating of the sample, i.e., the rate of weight loss. According to the thermal decomposition curve of the original P4VP sample, in the temperature range of 50–170 °C, the mass slowly decreases, with a weight loss of 5%. The sorption process increases the thermal stability of the material and reduces the rate of mass loss, which improves its structural properties. This indicates the improved stability. The maximum rate of weight loss for the original gP4VP has been recorded at 210 °C. At 550 °C, the anion exchange resin has mostly decomposed, and the process stabilized. Before and after the sorption at 200–250 °C, no new exothermic processes are observed, which confirms the presence of significant changes in the structure of the material.

At temperatures above 500 °C, the mass change slows down, indicating the formation of a stable residue. This residual mass consists of carbonaceous products from the polymer matrix decomposition along with the adsorbed gold and iron species, which remain non-volatile under these experimental conditions. Thus, the sorption results in an increased thermal stability of the P4VP anion exchanger, reduced mass loss, and a slower rate of thermal decomposition.

## 3. Materials and Methods

### 3.1. Materials

In this study, the laboratory-synthesized ion-exchange polymers were used as sorbents: polymethacrylic acid (PMAA) as a cation exchanger and poly-4-vinylpyridine (P4VP) hydrogel as an anion exchanger. Polymethacrylic acid-based hydrogels were synthesized in the presence of N,N-methylene-bis-acrylamide (Sigma-Aldrich, Darmstadt, Germany) as a cross-linking agent and a K_2_S_2_O_8_-Na_2_S_2_O_3_ (Sigma-Aldrich, Darmstadt, Germany) redox initiator system.

The process for producing poly-4-vinylpyridine hydrogels is based on cross-linking the linear polymer P4VP. The hydrogels were synthesized in an organic solvent, dimethylformamide (DMF, ≥99.8%, Sigma-Aldrich, Darmstadt, Germany). Epichlorohydrin (ECH, 99%, Sigma-Aldrich, Darmstadt, Germany) which forms chemical bonds between P4VP macromolecules and enables the formation of a three-dimensional polymer network, was used as a cross-linking agent.

This reaction produces a P4VP hydrogel with a high water absorption capacity and a sparsely bonded structure. Potassium dicyanoaurate (I) (K[Au(CN]_2_, Sigma-Aldrich, Darmstadt, Germany) and calcium hexacyanoferrate(II) trihydrate (K_4_[Fe(CN)_6_]·3H_2_O, Sigma-Aldrich, Darmstadt, Germany) were used to prepare the model solution. The concentrations of gold and iron ions in the resulting solution were strictly controlled throughout the experiment to ensure accuracy. All sorption experiments were conducted in distilled water at room temperature.

### 3.2. Preparation of Intergel Systems

**Figure 10:**
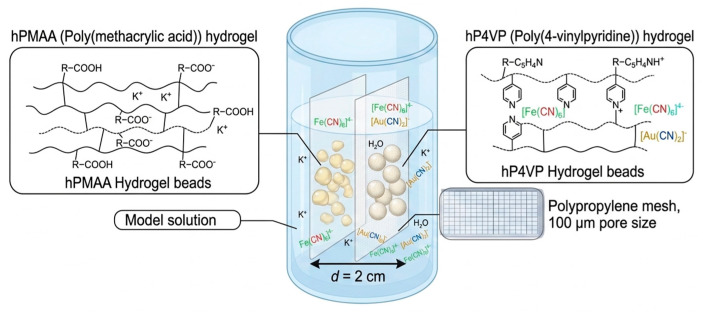
Illustration of the experimental setup for the hPMAA:hP4VP intergel system in an aqueous medium.

To facilitate the remote interaction between the two hydrogels, each polymer was placed into individual polypropylene mesh enclosures (pore size 100 μm). These meshes allow for the free diffusion of ions and solvent molecules while physically separating the polymer networks at a fixed distance of 2 cm within a glass beaker containing the model solution (Figure 10). This experimental setup ensures that the sorption process is governed by the long-range intergel effect rather than direct physical contact. The molar ratios of the hydrogels were varied as 6:0, 5:1, 4:2, 3:3, 2:4, 1:5, and 0:6, while keeping the total amount of the polymer material constant at 6 moles in each experimental setup.

### 3.3. Equipment

Samples were weighed using an HR-100AZG analytical balance (A&D Company, Ltd., Tokyo, Japan) with high precision. The residual concentrations of gold and iron ions in the solution were determined using an inductively coupled plasma atomic emission spectrometer (ICP-OES, iCAP PRO XP, Thermo Fisher Scientific, Loughborough, Leicestershire, UK). Measurements were performed in the wavelength range of 167.021–852.145 nm with a measurement error of less than 1%. The intensity of the spectral lines was used to quantify the concentration of each element. The functional groups and coordination bonds of the intergel system were identified using Fourier transform infrared (FTIR) spectroscopy. Spectra were recorded in transmission mode in the 500–4000 cm^−1^ range using a Nicolet 5700 spectrometer (Thermo Fisher Scientific, Madison, WI, USA). Samples were prepared as KBr pellets in a 1:50 mass ratio after thorough grinding to a particle size of less than 50 μm. The spectral resolution was 4 cm^−1^, and 32 scans were performed for each spectrum to improve the signal-to-noise ratio. The thermal stability and degradation of the PMAA and P4VP polymers before and after sorption were studied using a TG 209 F3 instrument (NETZSCH, Selb, Bavaria, Germany). Analysis was performed in the temperature range of 35–800 °C with sample weights ranging from 3.38 to 4.72 mg. Mass changes and thermal effects were recorded to evaluate the structural integrity of the sorbents.

### 3.4. Experimental Procedures

In our experiment, a model solution containing gold and iron ions was prepared by dissolving potassium dicyanoaurate (K[Au(CN]_2_) and potassium hexacyanoferrate(II) trihydrate (K_4_[Fe(CN)_6_]·3H_2_O) at a concentration of 30 mg/L for each ion. This solution was used to evaluate the sorption efficiency of the interpolymer system. The sorption experiments were performed using a total solution volume of 200 mL. At the specified time intervals (1 h, 2.5 h, 6 h, 24 h, and 48 h), 2 mL aliquots were taken from the solution to analyze the ongoing sorption process. Since the volume of each aliquot (2 mL) was only 1% of the initial solution volume, its impact on the concentration and the solid-to-liquid ratio was negligible. A total of 35 aliquots were collected, allowing for a detailed analysis of the sorption dynamics and the determination of the optimal time for the maximum sorption of gold and iron ions.

The degree of the polymer sorption (η) was calculated using the following formula:

(1) $$\eta =\frac{C_{o}-C_{e}}{C_{o}}\times 100\%,$$

where $C_{o}$ is the initial concentration of metal ions in the solution, mg/L; $C_{e}$ is the residual concentration of metal ions in the solution, mg/L.

Determining the degree of binding of gold and iron ions to the polymer chains involves a quantitative determination of the ionization degree of the macromolecule, as well as the degree of binding of the polymer structure and chains to gold and iron ions. The degree of binding of the intermodal units of the polymer chain to metal ions was calculated, using the following formula:

(2) $$\theta =\frac{\nu_{sorb}}{\nu }\times 100\%,$$

where $\nu_{sorb}$ is the amount of the sorbed gold and iron ions, in mol; ν is the polymer mass (if there are two polymers in the solution, it is calculated as the sum of the masses of each), in mol.

The distribution coefficients (Kd, mL·g^−1^) for Au(I) and Fe(II) ions were calculated from the equilibrium concentrations using the following equation:

(3) $$Kd=\frac{C_{o}-C_{e}}{C_{e}}\cdot \frac{V}{m}$$

where $C_{o}$ and $C_{e}$ (mg·L^−1^) are the initial and equilibrium concentrations of the metal ions, V (mL) is the volume of the solution, and m (g) is the mass of the dry sorbent.

The selectivity coefficient (β) was calculated as:

(4) $$\beta =\frac{{Kd}_{Au}}{{Kd}_{Fe}}$$

These parameters were used to evaluate the preferential uptake of Au(I) and Fe(II) ions by the PMAA–P4VP interpolymer system.

### 3.5. Statistical Analysis

All sorption experiments were performed in triplicate to ensure reproducibility. The results are presented as the mean values ± standard deviation (SD), with the relative standard deviation not exceeding 2%, indicating high precision of the experimental measurements. Data treatment and statistical analysis, including the calculation of means and SD, were carried out using OriginPro 2024 software (OriginLab Corporation, Northampton, MA, USA).

## 4. Conclusions

The results of this study have shown that the intergel systems based on polymethacrylic acid hydrogels (gPMAA) and poly-4-vinylpyridine (gP4VP) effectively sorb iron ions due to the long-distance interaction. According to the atomic emission spectroscopy data, it has been found that at the gPMAA cation exchanger ratio of 6:0, the sorption of iron ions is higher than the sorption of gold ions. Initially, the sorption of iron ions is 88.4%, but after 6 h this figure has increased up to 90.1%. However, the degree of the gold ion sorption has shown a significantly lower result: 3.7% (after 1 h) and 4.6% (after 6 h). At the 3:3 ratio of the gPMAA-gP4VP intergel system, the sorption of gold and iron ions has shown the maximum values, respectively: iron—91.20%, gold—65.67%. The results show that the system exhibits a high selectivity for iron ions only under the cation exchange conditions, while the gold ion sorption is minimal. The selective sorption of iron ions by the gPMAA-gP4VP intergel system, while gold ions remain in the solution, demonstrates the system’s high efficiency for the selective separation of these metals. This allows for the effective recovery and concentration of gold from complex gold-iron mixtures.

The results of the thermogravimetric analysis (TGA) and differential thermal analysis (DTA) have shown that the thermal stability of the sorbents gPMAA and gP4VP increases after the sorption due to the formation of the polymer–metal complexes that are resistant to thermal decomposition in the polymer structure.

## Figures and Tables

**Table 1:** Distribution coefficients (Kd) for Au(I) and Fe(II) ions and separation coefficients (β) at different molar ratios of the PMAA-P4VP intergel system.

Molar Ratio (PMAA-P4VP)	Kd(Au), mL/g	Kd(Fe), mL/g	β (Kd_Au_/Kd_Fe_)
6:0	105.8	15,456.6	0.0068
5:1	862.4	16,783.5	0.0514
4:2	1384	14,944.6	0.0926
3:3	2958.7	16,920.7	0.1749
2:4	2104.1	14,106.2	0.1492
1:5	2762	15,877.2	0.174
0:6	1516	13,765.4	0.1101

## Data Availability

The data presented in this study are available upon request from the corresponding author.
